# Neuroprotective effect of L-deprenyl on the expression level of the Mst1 gene and inhibition of apoptosis in rat-model spinal cord injury

**DOI:** 10.22038/IJBMS.2022.58031.12894

**Published:** 2022-01

**Authors:** Alireza Abdanipour, Ali Nikfar, Mahsa Nikbakht Rad, Iraj Jafari Anarkooli, Mojdeh Mansouri

**Affiliations:** 1 Department of Anatomy, School of Medicine, Zanjan University of Medical Sciences, Zanjan, Iran; 2 Department of Genetics and Molecular Medicine, Faculty of Medicine, Zanjan University of Medical Sciences, Zanjan, Iran

**Keywords:** Apoptosis, Bcl-2, Contusion, L-deprenyl, Mst1, Nrf2, Selegiline

## Abstract

**Objective(s):**

After primary tissue damage as a result of spinal cord injury (SCI), there is a period of secondary damage, which includes several cellular and inflammatory biochemical cascades. As a novel pro-apoptotic kinase, *Mst1* (serine/threonine kinase 4) promotes programmed cell death in an inflammatory disease model. This study aimed to evaluate *Mst1* gene expression levels in rats with spinal cord injury treated with L- deprenyl.

**Materials and Methods:**

The rats were divided into control (contusion), laminectomy, sham-operated (contused rats received 1 ml normal saline intraperitoneal), and treatment (contused rats received 5 mg/kg of L-deprenyl intraperitoneal; once a day for 7 days). The BBB (Basso, Beattie, and Bresnahan) scales were performed to assess motor function following SCI. Rats were sacrificed 28 days after SCI and the spinal cord lesion area was removed. Apoptosis and cavity formation in the spinal cord were determined by H&E staining and TUNEL assay, respectively. The mRNA levels of the *Mst1*, *Nrf2*, *Bcl-2*, and *PGC1α* genes were analyzed using real-time quantitative PCR.

**Results:**

The results showed significant improvement in motor function in the L- deprenyl group compared with the untreated group. Histological analysis showed a significant reduction in the number of tunnel-positive cells after injection of L-deprenyl, as well as a decrease in the volume of the cavity. In addition, L-deprenyl treatment increased the expression of the *Nrf2*, *Bcl-2*, and *PGC1α* genes, while reducing the expression of the *Mst1* gene in the spinal nerves.

**Conclusion:**

These results suggest that L-deprenyl is a promising treatment for spinal cord injury.

## Introduction

Despite advances in medical and surgical procedures, treating spinal cord injuries is still considered a significant challenge (1). Trauma to the spinal cord leads to a series of molecular and cellular events in the acute phase of the injury, which ultimately leads to the massive death of nerve cells (necrosis and apoptosis) in the chronic phase (2). *Mst1* is one of the main pro-apoptotic genes that stimulate the activation of caspase-3 (3). The pro-apoptotic functions of *Mst1* and *Mst2* have been known for a long time, although only their physiological cell substrates and their signaling cascades are determined. *Mst1* participates in apoptotic induction that enters the nucleus after caspase-mediated incision and induces chromatin condensation through DNA fragmentation (4). L-deprenyl is a type B (MAO-B) monoamine oxidase inhibitor and it was first used to treat Parkinson’s disease (5). The anti-apoptotic activity of L-deprenyl was also found **(**6**)**. Although the mechanism of action is not well known, L-deprenyl exerts its neuroprotective effect by protecting mitochondria by controlling antioxidant enzymes and anti-apoptotic genes (7, 8). Recent studies show that the activators *Nrf1*, *Nrf2* and *Bcl-2* *family* of co-activators control mitochondrial transcription specific factors. There have been many studies investigating the role of *Nrf2* in resistance to oxidative stress. The predominant cellular defense mechanism against oxidative stress is the Nrf2-Keap1 signaling pathway, followed by cytoprotective protein expression induction (9). The *Nrf2* gene influences cell proliferation, cell growth and, cell metabolism regulation through the phosphatidylinositol-3-kinase/AKT pathway and increases anti-apoptotic protein expression, *Bcl-2 *(10, 11). The use of biomolecules or protective agents to reduce the genes that promote apoptosis is a strategy to reduce the death of nerve cells (apoptosis/necrosis). In a rat model of spinal cord injury, we decided to assess the effect of L-deprenyl on *MST1* expression due to efficiency of L-deprenyl in enhancing motor function and reducing cell death.

## Materials and Methods

***Research study design***

In this experimental study, 40 female Wistar rats (weighing 250–300 g) were used. Animals with a light-dark cycle (12:12 hr) and unrestricted access to food and water were housed under normal humidity and temperature conditions. The experiment protocol was approved with the code of ZUMS.REC.1395.221 by the Animal Ethics Committee in accordance with the guidelines for the care and use of laboratory animals prepared by Zanjan University of Medical Science (ZUMS). Four experimental groups (n=10 per group) were divided into control (contusion), laminectomy, sham-operated (contused rats received 1 ml normal saline intraperitoneal), and treatment (contused rats received 5 mg/kg of L-deprenyl intraperitoneal; once a day for 7 days).

***Weight-drop contusion animal models***

Intraperitoneal (IP) injections of ketamine (80 mg/kg) and xylazine (10 mg/kg) were used to anesthetize the animals. Dorsal laminectomy at the level of T12/L1 was performed after shaving the hair around the thoracolumbar spine using an electric clipper. A metal rod of 10 g and about 2 mm in diameter was dropped on the exposed spine at a height of 25 mm, causing severe contusion (10). Animals recovered after surgery by subcutaneous injection of 2 ml of Ringer’s lactate solution (twice daily). Post-operative treatment for the first few days included manual bladder expression (twice daily) and intramuscular injection of 50 mg/kg cefazolin (Jabir Ibn Hayan, Tehran, Iran).

***Behavioral assessment***

Locomotor functions of the hind limb were assessed with the Basso Beattie Bresnahan (BBB) test from 0 to 21 (from complete paralysis to normal). The rats were recorded in a free field plastic container with a diameter of 110 cm and a height of 50 cm for four minutes with digital video cameras on days 1, 3, and 7 postoperatively and weekly for up to 4 weeks after surgery (12). 

***Histological analyses***

Under deep anesthesia, postoperative rats were sacrificed after 28 days and then transcardially perfused with heparin-containing saline for 0.5 min, followed by 4% paraformaldehyde in PBS for 5 min for histological assessments. The region of the lesion was carefully removed and the same fixation was then fixed for the next 12 hr. The tissues were automatically processed through a processor (Leica TP1020) and then placed in paraffin blocks. Serial sections of the spinal cord (10 μm thick) were prepared and chloroform dewaxed, rehydrated, then hematoxylin and eosin-stained (H & E). The volume of the cystic cavity (mm^3^) was then measured at a length of 4200 μm for the injured spinal cord (including the rostral and caudal regions of the injury epicenter) using the software ImageJ 1-44 and the Cavalieri method (equation 1, a: measured area, d: intersection distance)(13). Equation 1: 

DNA fragmentation was assessed in situ by an apoptosis detection kit (Roche, Germany). In brief, the paraffin-embedded pieces were deparaffinized and rehydrated into graded alcohol. The slides were microwave-pretreated for 10 min in a 10 mM citrate buffer (pH 6.0). Then they were incubated for 10 min with the blocking solution (3% H2O2 in methanol, Merck, Germany). The specimens were incubated with TUNEL reaction mixtures (terminal deoxynucleotidyl transferase and nucleotide mixtures in reaction buffer) at 37 °C for 60 min after washing in phosphate-buffered saline (PBS). The slides were stained with Converter-POD (anti-fluorescein antibody, sheep’s Fab fragment, and horse-radish peroxidase-POD conjugate) at 37 °C for 30 min after washing. The DAB substrate (Sigma-Aldrich, Germany) was added as chromogen, and hematoxylin was counterstained. Image J software quantified the percentage of TUNEL-positive cells in each region, and an average value was determined. Brown nuclear staining shows positive cells with apoptosis characteristic of fragmented nuclear chromatin. For each group, 10 sections were calculated for the number of stained cells in the rostral, central and caudal regions of the lesion areas.

***Real-time RT-qPCR***

RT-QPCR was performed using cDNA extracted from control and experimental groups. TRIzol ® (Invitrogen / Life Technologies) was used to isolate the complete RNA from injured spinal cord tissues. To synthesize 20 μl of cDNA according to the Revert Aid^TM^ First Strand cDNA Synthesis Kit (Fermentas, Germany), we used 1,000 ng of purified RNA. The cDNA was then used to quantify mRNA levels of *Mst1*, *Nrf2*, *Bcl-2*, and *PGC1α*. As an internal control for normalization, Glyceraldehyde 3-phosphate dehydrogenase (*GAPDH*) was used. RT-qPCR was performed by means of primers shown in Table 1. The PCR solution contained forward and reverse primers (200 nM each), cDNA (0.5 µl), SYBR® Green I (6.5 µl; Fermentas; Thermo Fisher Scientific, Inc.) and nuclease-free water up to the final volume of 12.5 μl. The PCR reaction was repeated for 40 cycles, each cycle including 15 sec at 95 °C followed by 1 min at 60 °C. The relative changes in the target mRNA levels were determined using the ΔΔCq method (14, 15).

***Statistical analysis***

Statistical analysis was performed with SPSS version 15 software. All data are presented from independent experiments, repeated three times the mean standard error of the mean (SEM). For data comparison between classes, one-way ANOVA and Tukey’s *post hoc* test were used. Data with a *P*-value less than 0.05 were considered significant.

## Results

***BBB score***

**Figure 1:**
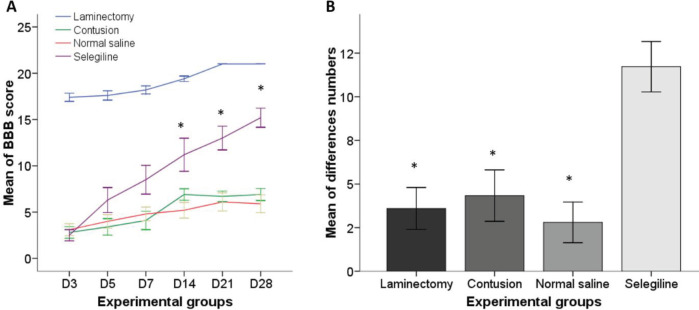
Evaluation of the Basso-Beattie Bresnahan (BBB) locomotive in the open field. BBB scores were calculated from day 3 to day 28 (6 time points) after injury. (A) Post-hoc one-way ANOVA at 14, 21, and 28 days post-SCI showed significant differences in the L-deprenyl treatment group compared with other experimental groups. (B) Bar graph shows the numerical difference (delta number) of the BBB score from 3 days to 28 days after the injury. The bar chart shows the mean ± SEM (n=8) at each point in time. * *P*<0.005

Scores of the hind limbs were recorded 3, 5, 7, 14, 21, and 28 days after SCI. In a spinal cord injury model, this test was performed to assess motor function. The behavioral study was performed in two phases, a preliminary test and the main test. In the preliminary test, all rats received 21 BBB points (normal movement). Statistically significant differences between groups were determined using one-way ANOVA (*P*<0.001). A post-Tukey test revealed a significant differences between the L-deprenyl treated group compared with untreated, vehicle and laminectomy groups on the days of 14 (treated: 10.66±1.40, untreated: 6.50±0.79, vehicle: 5.20±1.14, laminectomy: 19.40±0.40), 21 (treated: 12.66±0.98, untreated: 6.50±0.65, vehicle: 6.10±1.32, laminectomy: 21.00±0.00), and 28 (treated: 14.55±0.85, untreated: 6.83±0.71, vehicle: 5.90±1.28, laminectomy: 21.00±0.00) after injury (Figure 1A). The numerical difference (delta number) between 3 days and 28 days after the injury was significantly different between the L-deprenyl treated group (11.72±0.72) and the other groups (untreated: 6.33±0.73, vehicle: 2.80±0.58, laminectomy: 3.60±0.60) (Figure 1B). 

***Cavitation analysis***

**Figure 2:**
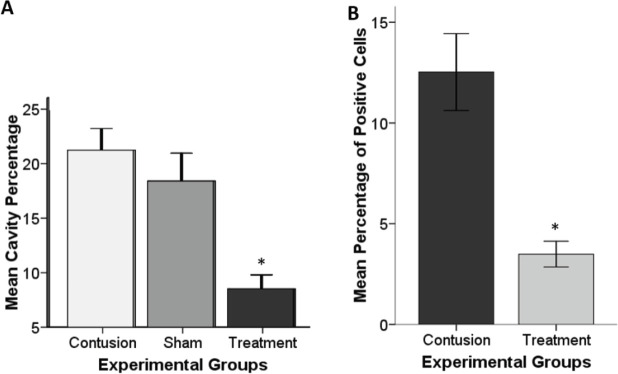
Percentage examination of the cavity 28 days after surgery at spinal length 4200 μm (including the rostral and caudal regions of the injury epicenter). (A) Bar graph demonstrates significant differences between L-deprenyl treated with contusion and sham groups. (B) Histogram of TUNEL-positive pixels at the lesion site of contused spinal cord experimental groups using brown pixel densitometry (mean percentage of apoptotic cells). Bars show the mean ± standard mean error; * *P*≤0.05 vs contusion group

**Figure 3:**
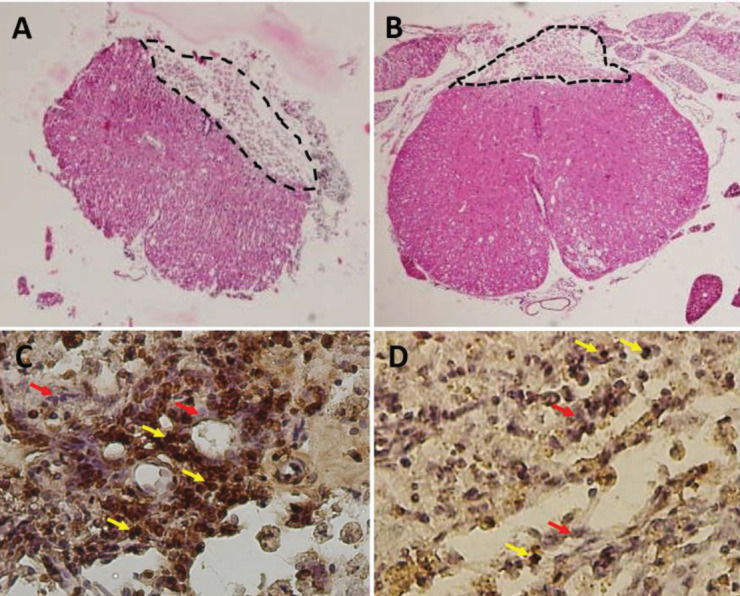
Representative H&E photomicrographs of the spinal cord showing the epicenter lesion area. Massive gray matter destruction and epicenter tissue loss were observed in the contusion group (A) compared with the L-deprenyl group (B). Black lines indicate cavity areas. (C, D) TUNEL positive immunoreactivity at the lesion site of the contused spinal cord of experimental groups on day 28 post-injury (C; contusion, D; L-deprenyl). Photomicrographs showing a decrease in the density of positive TUNEL cells in the group treated with L-deprenyl. The brown colors are immunopositive and the blue ones are stained with hematoxylin. The red-yellow arrows represent positive and normal cells, respectively. X400 magnification

The cavity area was measured as a mean percentage of the spinal cord length of 4.2 mm (Figures 2 and 3). To determine the therapeutic efficacy of L-deprenyl, the rats were sacrificed under deep anesthesia (28 days postoperatively) and a cavitation analysis was conducted. The results showed that the cavity volume was significantly reduced in the L-deprenyl treated group compared with the untreated groups (Figures 3A, B). Most of the cavity is located at the epicenter of the lesion. There was a significant difference between the groups treated with L-deprenyl (8.51 ± 0.64) and the untreated (21.23 ± 0.99) groups (Figure 2A). 

***Detection of apoptosis cells***

As a consequence of the TUNEL-assay, L-deprenyl has neuroprotective and anti-apoptotic effects. The percentage of apoptotic cells was calculated at 28 days post-injury in three regions (rostral, central, and caudal) of the lesioned area**. **A significant difference was found in the L-deprenyl-treated group (3.48 ± 0.31) compared with the non-treated contusion group (12.53 ± 0.95, *P*<0.05). These findings were shown in Figure 2B and Figures 3C, D.

***Gene expression***

**Figure 4:**
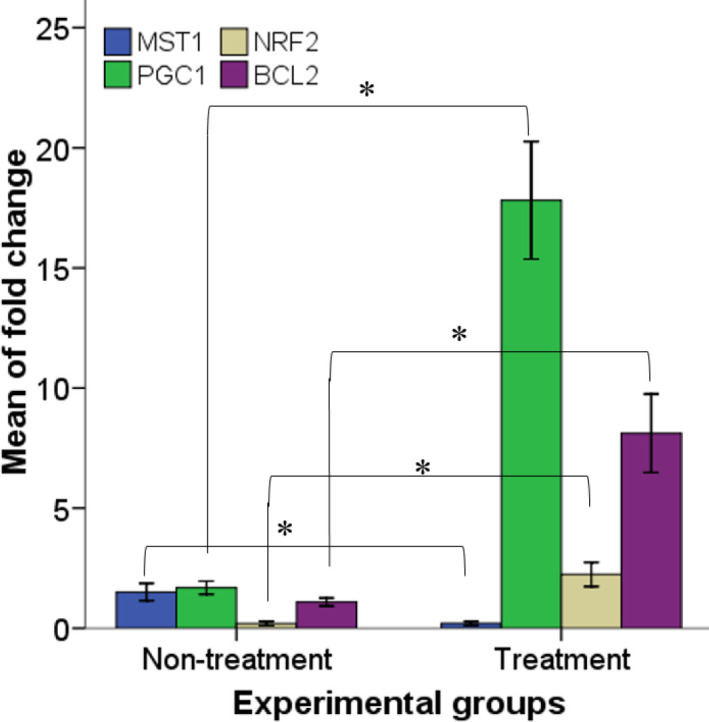
Real-time RT-PCR quantitative results, compared with the laminectomy group, normalize GAPDH mRNA amplification. The bars show the mean ± SEM; * (Compared with the untreated group, *P*<0.05)

The results of gene expression were presented with reference to the control (laminectomy) group (Figure 4). The results showed that L-deprenyl treatment substantially improved *PGC1α*, *Nrf2,* and *Bcl-2* mRNA levels (17.81 ± 1.22, 2.24 ± 0.0.25, and 8.11 ± 0.81; respectively) as compared with the non-treatment group (1.69 ± 0.13, 0.19 ± 0.04, and 1.08 ± 0.08; respectively for *PGC1α*, *Nrf2,* and *Bcl-2*). A significant increase in *Mst1* (1.51 ± 0.17) mRNA was also found in the untreated group compared with the L-deprenyl treatment group (0.2 ± 0.03).

**Table 1:** Primer sequences and PCR parameters. Primers for amplification of target sequences and their Gen Bank accession numbers

Reference sequence	Sequence	Gene name
NM_001107800.1	F: GCTAAAGTGAAGTGGACGGATACC	*Mst1*
	R: GGAACAGTTGCTACCAGAGTGTCAG	
NM_031789.2	F: CACCAGTGGATCTGTCAGCTACTC	*Nrf2*
	R: GTGGTGAAGACTGAGCTCTCAACG	
NM_017008.4	F: GTGGCCTTCTTTGAGTTCGGTG	*Bcl-2*
	R: TTGTCAGCAATGCATCCTGCAC	
NM_031347.1	F: GACAGAACTGAGAGACCGCT	*PGC1α*
	R: ATTCCCGAGTTACAGTGTCT	
NM_017008.4	F: TTGTCAGCAATGCATCCTGCAC	*GAPDH*
	R: GTCTGGGATGGAATTGTGAG	

## Discussion

The results of this study show that L-deprenyl in the rat contusion model was effective in treating spinal cord injury, significantly improved motor function, and reduced cavity size compared with the untreated group. Furthermore, the results of the TUNEL test show that L-deprenyl can reduce apoptosis in treated rats. Similarly, the use of real-time PCR has shown that L-deprenyl therapy increases the expression of the three anti-apoptotic genes *Nrf2*, *Bcl-2*, and *PGC1α* and decreases the expression of the pro-apoptotic *Mst1* gene. Spinal cord injury is a two-step procedure that involves the primary mechanical injury followed by inflammation and apoptosis (16). Secondary damage is characterized by further destruction of neurons and glial cells, leading to damage spreading to higher segments (17). The results of this study showed that damage to the spinal cord causes apoptosis at the site of injury and adjacent areas. In addition, histological evaluation showed that a large cavity was formed in the center of the lesion as a result of the death of a significant number of nerves and glia that could spread the lesion up and down. Identifying mechanisms that promote or prevent neuronal inflammation and apoptosis, and new approaches to the prevention and treatment of neurodegenerative diseases are emerging (10). Rasagiline and L-deprenyl are two monoamine oxidase B (MAO-B) inhibitors. These inhibitors have neuroprotective effects on monoamine oxidases A and B. The role of MAO-B in the apoptotic process was not well known, but MAO-A was involved in mediating the expression of anti-apoptotic genes such as *Bcl-2* (18). Tatton *et al.,* first reported in 1996 that deprenyl at low concentrations reduced apoptosis in neurons. L-deprenyl acts on gene expression to preserve mitochondrial activity and to suppress cytoplasmic oxidative radical levels and thereby minimize apoptosis. They found that L-deprenyl had an effect on the growth of some neurons at low doses and reduced oxidative radicals by inhibiting the monoamine oxidase B enzyme in injured cells, thereby reducing apoptosis (19). Recent findings on neural stem cells also show that L-deprenyl enhances the viability of neural stem cells *in vitro* under oxidative damage conditions (20). According to Maruyama *et al.*, L-deprenyl suppresses apoptotic death in nerve cells by modifying intracellular activities. Researchers have demonstrated that L-deprenyl and its related compounds can reduce the rate of apoptotic cell death in the aging population and prevent neurodegenerative diseases (21). Results from studies showed that after the use of L-deprenyl, more than 51 percent of damaged cells in brain infarction survived (22). L-deprenyl and its metabolites can cause apoptosis at high concentrations due to dopaminergic effects, but low concentrations may reduce apoptosis by reducing adverse effects and preventing neuronal death (23). Researchers have already observed the molecular mechanisms involved in reducing L-deprenyl-based apoptosis. There are detailed findings on the activation of apoptosis mechanisms after spinal cord injury (24, 25). Regarding the role of apoptosis in spinal cord injury, new therapies, such as inhibition of genes involved in apoptosis signaling pathways such as Mst1, can lead to a decrease in cell death (26, 27).* Mst1* is considered to be one of the proteins specifically and indirectly associated with caspase-3, which can be triggered by stress and apoptosis, and a wide variety of responses are involved (28, 29). In this study, we show that after SCI, L-deprenyl decreased *Mst1* gene expression and apoptosis rate *Mst1* can affect several biological processes, in addition to cell death in neurons (30). Consistent with this interpretation, *Mst1* -FOXO signaling promotes lifespan and healthspan in Caenorhabditis elegans (31). Overexpression of *Mst1* induces apoptotic changes such as chromatin condensation, and *Mst1* is required for apoptosis induced by certain genotoxic agents, including UV and staurosporine (32). During apoptosis, a commonly observed specific histone modification is the phosphorylation of histone H2B at Ser-14, and *Mst1* has been identified as the activating kinase (33).* Mst1* plays an important role in apoptosis mediation, but its specific role has not been well defined (4, 34). *In vivo* reports have shown that the absence of *Mst1* improved spinal motor neuron survival following trauma, locomotive ratings, and synapse survival (35). It is specifically activated in post-traumatic spinal motor neurons and its deficiency can correct the dysfunction of the autophagy-lysosome pathway in injured motor neurons via more autophagosome formation and enhancement of autolysosome degradation, through which the adequate degradation of toxic protein aggregates reduced the motor neuron loss, and eventually, promoted synapse survival and improved behavioral outcome(36). No experiments have yet been performed to investigate the effects of L-deprenyl on *Mst1* gene expression. Researchers showed that *Mst1* activity was increased presymptomatically in motor neurons, but not in glial cells, of the spinal cord in mice and that deficiency of the MST1 gene improved the severity of disease manifestations in the ALS model mice (37). Researchers defined a critical oxidative stress pathway in neurons mediated by the c-Abl-* Mst1* complex. Identification of the signaling link between c-Abl and Mst1 kinases bridges the gap between oxidative stress and neuronal cell death dependent on activation of MST1, and also indicates a novel biological role for both kinases (38). *Mst1*-overexpression may induce memory impairments via disturbing the patterns of neural activities, which is possibly associated with the abnormal GABAergic expression level (39). The cells can be protected against apoptosis by another category of regulatory proteins, such as anti-apoptotic *Bcl-2*, *Bcl-XL*,* Nrf2*, and *PGC1α*. Several apoptotic signals converge on the activation of caspase, and *Bcl-2*, *Bcl-XL*, and *Nrf2* are also regulators of this pathway (40, 41). Mitochondria also have roles in signaling, cellular differentiation, cell growth, and cell death (42). The main regulators of mitochondrial biogenesis include the peroxisome proliferator-activated receptor-gamma coactivator (PGC) family of transcriptional activators, which consists of *PGC1α*, *PGC1β*, and PGC-related coactivator (PRC) (43). *PGC1α *plays a role in the activating of nuclear respiratory factor 2 and together they coactivate nuclear respiratory factor 1. Consequently, nuclear respiratory factor 1 activates Tfam, which is important for mitochondrial DNA (mtDNA) transcription, translation, and repair. Thus, *PGC1* family coactivators act as mediators between the environment and the transcriptional machinery regulating the biogenesis of mitochondria (44). *Nrf2* is one of the important factors involved in the prevention of apoptosis proteins. This transcription factor, with increasing expression of antioxidant and detoxifying enzymes, prevents cell death and is known as an anti-apoptotic factor (45). Studies suggest that Keap1 preserves *Nrf2* in the cytoplasm. Keap1 acts as a substrate adapter for *Nrf2* degradation mediated by Cul3/Rbx1 (46). In this study, our results indicated, expression of *Nrf2* increased in the L-deprenyl -treated group. L-deprenyl also enhanced the *Nrf2* ability to bind to the antioxidant response elements (AREs) sequence at the beginning of the oxygenase-1 gene and thus improved the expression of this enzyme (47). Therefore, L-deprenyl activates the *Nrf2*/*ARE* pathway, independently inhibits the enzyme monoamine oxidase B, and protects cells from MPP (+) -induced oxidative damage (48). Other researchers revealed that intraperitoneal L-deprenyl therapy in mice subjected to 3-nitropropionic acid resulted in decreased lipid peroxidation, caspase-3, and Bax, increased catalase and superoxide dismutase activity, and *Bcl-2* (49). Hasegawa et al., evaluating the effects of L-deprenyl on SH-SY5Y cells, observed that this drug increased *Bcl-2* at mRNA and protein levels via the enzyme monoamine oxidase B (50). The release of cytochrome c from mitochondria is regulated by *Bcl-2* family proteins (51). As anti-apoptotic enzymes, they can inhibit cell death by inhibiting the release of cytochrome c, physiological modulation of membrane permeability, release of calcium, and oxidative stress. In the previous research, it was shown that under conditions of oxidative damage, treatment of neuronal cells with L-deprenyl may reduce apoptosis and increase the expression of *Bcl-2* and Hspa4 genes (20, 52). In another study, we concluded that the expression of lovastatin *PGC1α* and *Nrf2* genes protects bone marrow nerve cells from oxidative damage and prevents apoptotic death (53). In the present study, we showed that L-deprenyl increases the mRNA levels of *PGC-1α*, *Nrf2*, and *Bcl-2*.

## Conclusion

The findings of this research revealed that in the rat spinal cord injury model, the use of L-deprenyl *in vivo* resulted in an increased recovery of motor function. L-deprenyl also protects nerve cells by inhibiting the neuroprotective effects of apoptosis. In addition, given the possible role of L-deprenyl in the expression of three *Nrf2*, *Bcl-2*, and *PGC1α *anti-apoptotic genes and the decrease in the expression of the pro-apoptotic *Mst1* gene, this drug may be considered an acceptable and low-cost candidate for spinal cord injury and other diseases of the nervous system.

## Authors’ Contributions

AA Designed the study, carried out the research project, analyzed the results, and wrote the manuscript; AN and MM were research collaborators; MNR was a research collaborator; I JA was an advisor.

## Conflicts of Interest

The authors declare that they do not have any conflicts of interest. We affirm that we have read the policy of the journal on issues related to ethical publishing and that this report is consistent with those guidelines.
